# TACE plus lenvatinib and tislelizumab for intermediate-stage hepatocellular carcinoma beyond up-to-11 criteria: a multicenter cohort study

**DOI:** 10.3389/fimmu.2024.1430571

**Published:** 2024-07-26

**Authors:** Song Chen, Tang Shuangyan, Feng Shi, Hongjie Cai, Zhiqiang Wu, Liguang Wang, Ping Ma, Yuanmin Zhou, Qicong Mai, Fan Wang, Jiaming Lai, Xiaoming Chen, Huanwei Chen, Wenbo Guo

**Affiliations:** ^1^ Department of Minimally Invasive Interventional Therapy, State Key Laboratory of Oncology in South China, Guangdong Provincial Clinical Research Center for Cancer, Sun Yat-sen University Cancer Center, Guangzhou, China; ^2^ Department of Interventional Radiology, The First Affiliated Hospital of Sun Yat-sen University, Guangzhou, China; ^3^ Department of Interventional Radiology, Guangdong Provincial People’s Hospital, Guangzhou, China; ^4^ Department of Hepatopancreatic Surgery, The First People’s Hospital of Foshan, Foshan, China; ^5^ Department of Oncology, The Twelfth People’s Hospital of Guangzhou, Guangzhou, China; ^6^ Center of Hepato-Pancreato-Biliary Surgery, The First Affiliated Hospital of Sun Yat-sen University, Guangzhou, China

**Keywords:** hepatocellular carcinoma, intermediate-stage, up-to-eleven criteria, transartrial chemoembolization, combination therapy

## Abstract

**Background:**

Intermediate-stage (BCLC-B) hepatocellular carcinoma (HCC) beyond the up-to-11 criteria represent a significant therapeutic challenge due to high and heterogeneous tumor burden. This study evaluated the effectiveness and safety of transarterial chemoembolization (TACE) in combination with lenvatinib and tislelizumab for these patients.

**Methods:**

In this retrospective cohort study, patients with unresectable intermediate-stage HCC beyond the up-to-11 criteria were enrolled and divided into TACE monotherapy (T), TACE combined with lenvatinib (TL), or TACE plus lenvatinib and tislelizumab (TLT) group based on the first-line treatment, respectively. The primary endpoint was overall survival (OS). The secondary outcomes included progression-free survival (PFS), tumor response according to RESIST1.1 and modified RECIST, and adverse events (AEs).

**Results:**

There were 38, 45, and 66 patients in the T, TL, and TLT groups, respectively. The TLT group exhibited significantly higher ORR and DCR than the other two groups, as assessed by either mRECIST or RECIST 1.1 (all *P*<0.05). Median PFS and OS were significantly longer in the TLT group compared with the T group (PFS: 8.5 vs. 4.4 months; OS: 31.5 vs. 18.5 months; all *P*<0.001) and TL group (PFS: 8.5 vs. 5.5 months; OS: 31.5 vs. 20.5 months; all *P*<0.05). The incidence of TRAEs was slightly higher in the TLT and TL groups than in the T group, while all the toxicities were tolerable. No treatment-related death occurred in all groups.

**Conclusions:**

TACE combined with lenvatinib and tislelizumab significantly improved the survival benefit compared with TACE monotherapy and TACE plus lenvatinib in patients with intermediate-stage HCC beyond the up-to-11 criteria, with an acceptable safety profile.

## 1 Introduction

Hepatocellular carcinoma (HCC) stands as the sixth most common malignancy and the third leading cause of cancer-related deaths worldwide (1). Ablation, liver resection, and liver transplantation are curative options for patients with HCC, but approximately 80% of the patients are diagnosed at the intermediate or advanced stage, and these curative strategies are unsuitable (2, 3). Transarterial chemoembolization (TACE) is the recommended standard of care for intermediate HCC, defined as Barcelona Clinic Liver Cancer (BCLC) stage B disease (4, 5). However, BCLC-B stage HCC is a very heterogeneous disease with a wide range of tumor burden and liver function, and not all patients can benefit from TACE (5, 6). It is worth noting that high tumor burden is an important component used by various subclassification or prediction models to select patients unsuitable for TACE (7).

In order to optimize prognosis and optimal treatment strategies, some studies have been conducted to develop a tailored subgroup stratification for BCLC-B stage HCC (8–11). For instance, Bolondi et al. (9) proposed the first subclassification for BCLC-B HCC based on the up-to-7 criteria in 2012, combining the number of tumors and the size of the largest tumor, with the sum being no more than 7. Subsequent studies have shown that the up-to-11 criteria (combining the number of tumors and the size of the largest tumor, with the sum being no more than 11) (12) may be more discriminative than the up-to-7 criteria for predicting survival after TACE. Still, the efficacy of TACE is limited in patients with high tumor burden, particularly those beyond the up-to-11 criteria (12, 13). The 7-11 criteria were also proposed, combining the number of tumors and the size of the largest tumor, with >11 being a heavy tumor burden, 7-11 an intermediate burden, and <7 a low burden (14). Moreover, there is a growing apprehension regarding the deleterious effects on hepatic function following repeated TACE procedures due to tumor progression or residual disease. Given these challenges, there is a pressing demand to explore TACE combination therapies that aim to improve therapeutic outcomes and reduce the number of TACE sessions. The theoretical synergy of TACE plus molecular targeted agents (MTAs) boasting anti-VEGF activity, such as sorafenib and lenvatinib, offers hope for improved prognosis. Regrettably, several early clinical randomized controlled trials (RCTs) comparing patient survival with combination therapy vs. TACE monotherapy have yielded negative results (15–17). None of the combination therapies are currently recommended, underscoring the great unmet need to explore novel combination strategies.

Recently, immune checkpoint inhibitors (ICIs) have shown promising efficacy and safety for advanced HCC. The phase III RATIONALE 301 trial demonstrated a clinically meaningful benefit in overall survival (OS) with tislelizumab monotherapy compared with sorafenib (18). In addition, the LEAP-002 trial examined the combination of lenvatinib plus pembrolizumab vs. lenvatinib alone in patients with unresectable HCC; although the trial did not reach a positive result, the OS and progression-free survival (PFS) were significantly longer in the combination group than in the monotherapy group (19). The CARES-310 trial showed that camrelizumab plus rivoceranib showed benefits in PFS and OS compared with sorafenib for patients with unresectable HCC (20). In addition, several RCTs confirmed the efficacy and safety of combining programmed death 1 (PD-1) or programmed death-ligand 1 (PD-L1) inhibitors with anti-VEGF antibodies or tyrosine kinase inhibitors (TKIs) in advanced HCC (21–23). As TACE is a locoregional inducer of immunogenic cell death in HCC, it can transform an immunosuppressive microenvironment into an immunostimulatory one, thereby promoting tumor-specific immune response and improving the response to ICIs (24). Besides, TACE combined with targeted therapy and immunotherapy has been gradually become a significant treatment strategy for HCC conversion (25). Nevertheless, few data are available regarding the triple combination therapy in patients with BCLC-B HCC beyond the up-to-11 criteria in clinical practice, who will have a heavier tumor burden (9, 12, 14) than the patients included in the previous RCTs that were mostly based on the up-to-7 criteria.

Therefore, this study aimed to evaluate the first-line treatment outcomes of TACE plus lenvatinib and tislelizumab in patients with BCLC-B HCC beyond the up-to-11 criteria compared with TACE monotherapy and TACE combined with lenvatinib. The results could contribute to developing effective treatment options for intermediate-stage HCC and provide a basis for future clinical trials.

## 2 Methods

### 2.1 Study design and patients

This multicenter retrospective cohort study included patients with unresectable BCLC-B HCC beyond the up-to-11 criteria. These patients underwent TACE between January 2016 and December 2022 at one of the four participating centers in China. The study was conducted in accordance with the Declaration of Helsinki (as revised in 2013). The study was approved by the ethics committee of the First Affiliated Hospital of Sun Yat-sen University (#2021-782), and individual consent for this retrospective analysis was waived. The study was reported according to the STROCSS criteria.

The inclusion criteria were 1) age between 18-75 years, 2) radiologically or pathologically diagnosed with HCC according to the practice guidelines of the American Association for the Study of Liver Diseases (26), 3) classified as BCLC-B or C stage beyond the up-to-11 criteria, with the sum of the diameter of the largest tumor (in cm) and the total number of tumors exceeding 11, 4) unresectable HCC according to the evaluation by a multidisciplinary team, 5) received TACE monotherapy, TACE plus lenvatinib, or TACE plus lenvatinib and tislelizumab as first-line treatment, 6) classified as Child-Pugh A or B before the first TACE procedure.

Patients were excluded if they had 1) other malignancies within 5 years before HCC diagnosis, 2) insufficient organ function or inadequate hematologic function, or 3) incomplete key medical data. Laboratory tests and imaging evaluations, including enhanced computed tomography (CT) and magnetic resonance imaging (MRI), were obtained within 1 week before the initial treatment.

### 2.2 Grouping

The patients were stratified into three distinct groups based on their first-line treatment regimen: T (TACE monotherapy), TL (TACE combined with lenvatinib), and TLT (TACE combined with lenvatinib and tislelizumab). The treatment strategy selection was determined based on the physician’s recommendation, the patient’s financial condition, and the accessibility of the targeted and immune drugs.

### 2.3 Standardized TACE procedure

The tip of the catheter was inserted into the tumor-feeding arterial branches according to tumor size, location, and vascular supply. Chemoembolization was performed utilizing an emulsion of doxorubicin and lipiodol, followed by introducing microspheres or an absorbable gelatin sponge. The embolization endpoint was classified according to the previously established subjective angiographic chemoembolization endpoint scale (SACE). Generally, the embolization endpoint corresponded to SACE levels III or IV, indicating diminished or absent antegrade arterial flow without tumor blush (27). All interventions were handled by the same physicians at each participating center with at least 10 years of experience in interventional radiology. Subsequent TACE sessions were administered as deemed necessary by the treating clinicians.

### 2.4 Lenvatinib treatment

Lenvatinib was initiated 3 to 5 days after the first TACE session, with dosage tailored to patient weight: 12 mg for those weighing above 60 kg and 8 mg for those below 60 kg. The dose was maintained in case of grade 1-2 adverse events (AEs), and supportive treatments were promptly introduced to manage the AEs. If grade 3-4 AEs occurred, the dose was reduced to 8 mg and 4 mg, respectively, or the frequency was reduced to once every other day until the AEs were resolved or alleviated. Persistent AEs led to dose suspension until they were alleviated or disappeared.

### 2.5 Tislelizumab treatment

For patients in the TLT group, tislelizumab was administered intravenously once every 3 weeks starting on the second day after TACE. Symptomatic treatment was provided to manage grade 1-2 AEs. If grade 3-4 AEs occurred, tislelizumab was suspended until they were resolved or alleviated. If grade 3-4 AEs recurred, tislelizumab was permanently discontinued. Dose adjustment for tislelizumab was not allowed.

### 2.6 Assessment and outcomes

A contrast-enhanced CT or MRI was performed every 4-6 weeks after TACE by two independent, experienced radiologists, and the interval was prolonged to 2-3 months if systemic maintenance therapy was given. The primary outcome was overall survival (OS). The secondary outcomes included progression-free survival (PFS), tumor response, and adverse events (AEs). Treatment response, objective response rates (ORRs), and disease control rates (DCRs) were determined according to the modified response evaluation criteria in solid tumors (mRECIST) and RECIST version 1.1. ORR was defined as the proportion of patients who achieved complete response (CR) or partial response (PR). DCR was defined as the proportion of patients who achieved CR, PR, or stable disease (SD). PFS was defined as the time from admission to disease progression (as per mRECIST) or death from any cause, whichever came first. OS was defined as the time from admission to death from any cause. Treatment-related AEs (TRAEs) were recorded and graded according to CTCAE version 5.0.

### 2.7 Theory/calculation

All statistical analyses were performed using R 4.0.3 (R Foundation Inc., Vienna, Austria) and SPSS 25.0 (IBM, Armon, NY, USA). Continuous variables were expressed as means ± standard deviations or medians (interquartile range [IQR]) and compared using Student’s t-test or the Mann-Whitney U-test. Categorical variables were presented as numbers and percentages and compared using the chi-squared test or Fisher’s exact test. The Kaplan-Meier method was used to analyze time-to-event variables, and the differences were examined using the log-rank test. Univariable and multivariable Cox regression analyses were performed to identify the factors associated with PFS and OS. Variables with *P ≤* 0.10 in the univariable analyses were included in the multivariable analysis. Subgroup analyses of PFS and OS were performed to analyze the superiority of TLT versus TL. Two-sided *P*<0.05 was considered statistically significant.

## 3 Results

### 3.1 Baseline characteristics of patients

**Figure 1:**
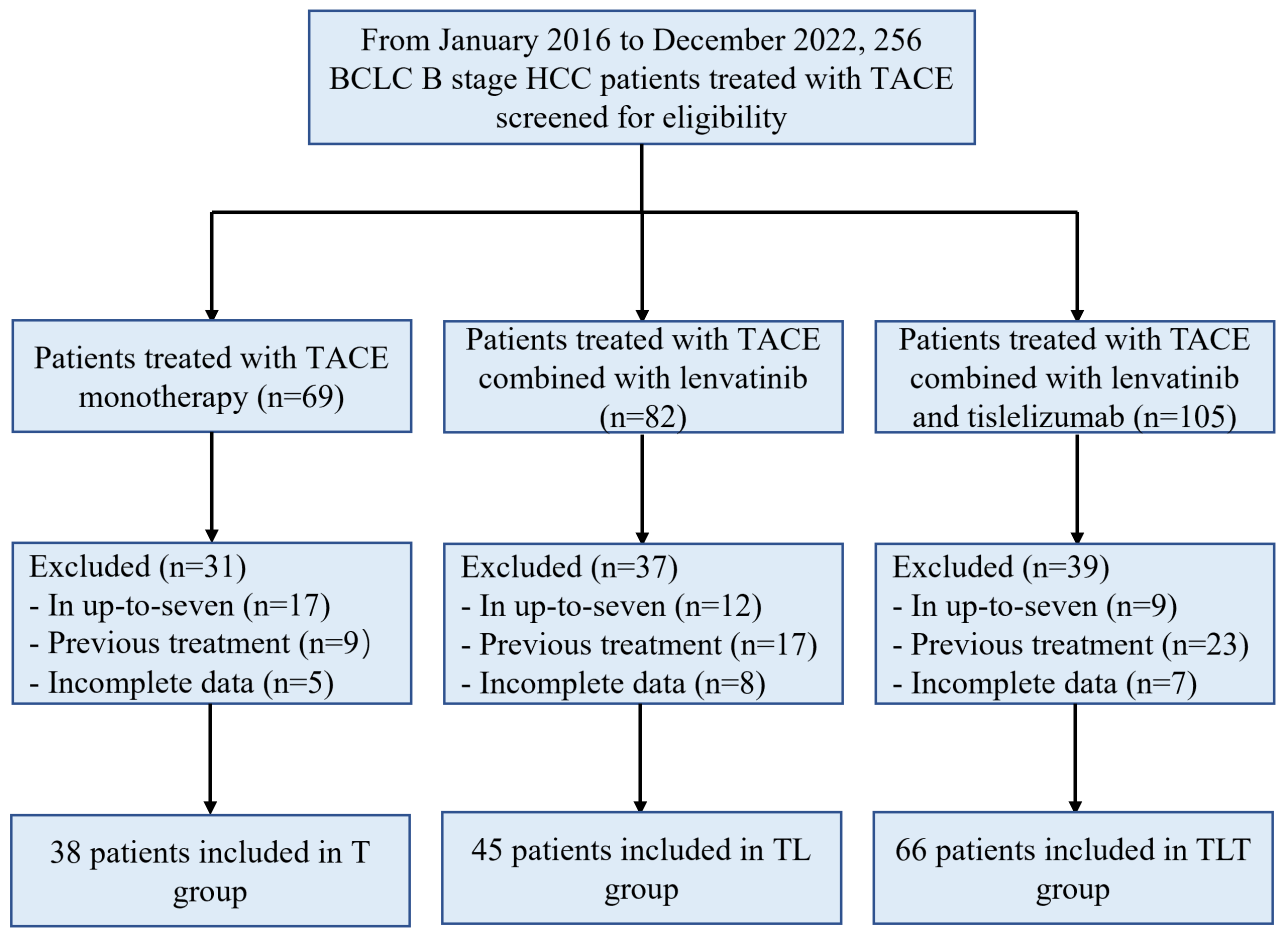
Study flowchart. T, TACE, transarterial chemoembolization; TL, TACE combined with lenvatinib; TLT, TACE combined with lenvatinib and tislelizumab; BCLC, Barcelona Clinic Liver Cancer.

A total of 256 patients were assessed for eligibility, and 107 were excluded. Finally, 149 patients were included: 38 in the T group, 45 in the TL group, and 66 in the TLT group (**Figure 1**). As it was a multicenter study, the numbers of patients provided by each participating center were 19/10/5/4 for the T group, 21/16/3/5 for the TL group, and 34/14/12/6 for the TLT group. There were no significant differences in baseline characteristics among the three groups (all *P*>0.05) (**Table 1**). The patients were 56.5 ± 13.0, 56.6 ± 12.1, and 55.8 ± 11.2 years, respectively, and 138 (92.6%) were males. Among the 149 patients, 124 (83.2%) patients had hepatitis B virus infection, and 120 (80.5%) had cirrhosis. The median number of TACE sessions was six (range, four to 11), four (range, one to eight), and three (range, one to six) in the T, TL, and TLT groups, respectively.

**Table 1:** Baseline characteristics of the patients.

Characteristics	T (n=38)	TL (n=45)	TLT (n=66)	*P*
Age (years)	56.5 ± 13.0	56.6 ± 12.1	55.8 ± 11.2	0.940
Sex				0.974
Male	35 (92.1)	42 (93.3)	61 (92.4)	
Female	3 (7.9)	3 (6.7)	5 (7.6)	
Etiology				0.474
Hepatitis B virus	34 (89.5)	36 (80.0)	54 (81.8)	
Others	4 (10.5)	9 (20.0)	12 (18.2)	
Child-Pugh class				0.716
A	32 (84.2)	40 (88.9)	59 (89.4)	
B	6 (15.8)	5 (11.1)	7 (10.6)	
Cirrhosis				0.982
Yes	31 (81.6)	36 (80.0)	53 (80.3)	
No	7 (18.4)	9 (20.0)	13 (19.7)	
Tumor size (cm)	5.9 (4.9-8.1)	5.8 (3.1-8.0)	6.0 (3.9-8.4)	0.613
>7	23 (60.5)	27 (60.0)	41 (62.1)	0.972
≤7	15 (39.5)	18 (40.0)	25 (37.9)	
Number of lesions				0.967
>3	34 (89.5)	40 (88.9)	58 (87.9)	
2-3	4 (10.5)	5 (11.1)	8 (12.1)	
AFP (ng/mL)				0.257
<400	22 (57.9)	28 (62.2)	48 (72.7)	
≥400	16 (42.1)	17 (37.8)	18 (27.3)	
Sessions of TACE	6 (4-11)	4 (1-8)	3 (1-6)	0.059

T, TACE, transarterial chemoembolization; TL, TACE combined with lenvatinib; TLT, TACE combined with lenvatinib and tislelizumab; AFP, α-fetoprotein.

### 3.2 Treatment response

According to mRECIST, the CR rates for the T, TL, and TLT groups were 0, 8.9%, and 16.7%, respectively (*P*=0.024). The ORRs were 31.6%, 53.3%, and 80.3% (*P*<0.001), and DCRs were 73.7%, 80.0%, and 93.9% (*P*=0.014) for the T, TL, and TLT groups, respectively. According to RECIST 1.1, the ORRs were 13.2%, 28.9%, and 45.5% (*P*=0.003), and DCRs were 65.8%, 80.0%, and 92.5% (*P*=0.003) in the T, TL, and TLT groups, respectively (**Table 2**).

**Table 2:** Tumor response rates according to mRECIST and RECIST 1.1.

Response, n (%)	T	TL	TLT	*P*	T	TL	TLT	*P*
	mRECIST				RECIST 1.1			
CR	0 (0)	4 (8.9)	11 (16.7)	0.024	0 (0)	0 (0)	0 (0)	
PR	12 (31.6)	20 (44.4)	42 (63.6)		5 (13.2)	13 (28.9)	30 (45.5)	
SD	16 (42.1)	12 (26.7)	9 (13.6)		20 (52.6)	23 (51.1)	31 (47.0)	
PD	10 (26.3)	9 (20.0)	4 (6.1)		13 (34.2)	9 (20.0)	5 (7.5)	
ORR	12 (31.6)	24 (53.3)	53 (80.3)	<0.001	5 (13.2)	13 (28.9)	30 (45.5)	0.003
DCR	28 (73.7)	36 (80.0)	62 (93.9)	0.014	25 (65.8)	36 (80.0)	61 (92.5)	0.003

mRECIST, modified Response Evaluation Criteria in Solid Tumors; RECIST 1.1, Response Evaluation Criteria in Solid Tumors 1.1; T, TACE, transarterial chemoembolization; TL, TACE combined with lenvatinib; TLT, TACE combined with lenvatinib and tislelizumab; CR, complete response; PR, partial response; SD, stable disease; PD, progressive disease; ORR, objective response rate; DCR, disease control rate; CI, confidence interval.

### 3.3 Survival outcomes and associated factors

**Figure 2:**
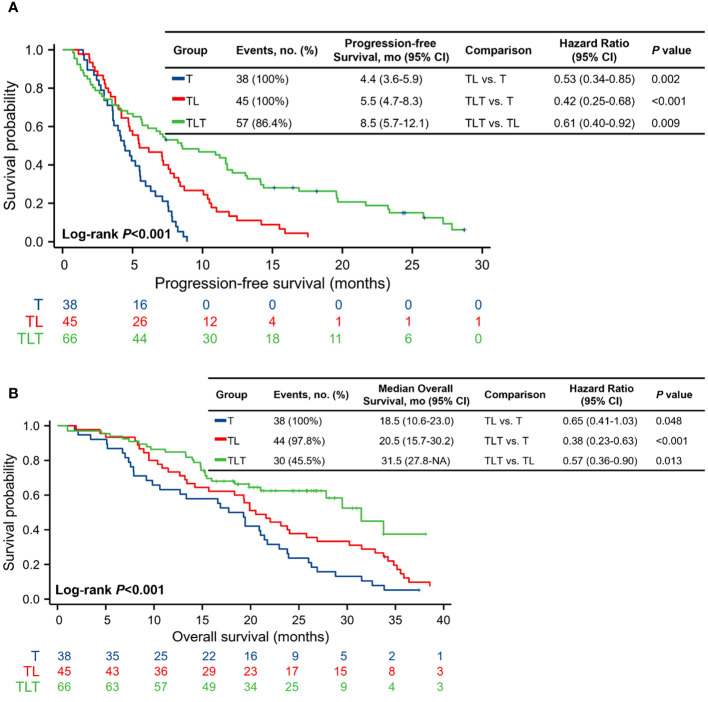
Kaplan-Meier analysis of progression-free survival. **(A)** and overall survival **(B)**. CI, confidence interval; T, TACE, transarterial chemoembolization; TL, TACE combined with lenvatinib; TLT, TACE combined with lenvatinib and tislelizumab.

As of the last follow-up on December 31, 2023, the median follow-up for all patients was 29.8 (range, 12.0-49.4) months. PFS was significantly longer in the TLT group (median, 8.5 [95% CI, 5.7-12.1] months) compared with the T (median, 4.4 [95% CI, 3.6-5.9] months; *P*<0.001) and TL (median, 5.5 [95% CI, 4.7-8.3] months; *P*=0.009) groups (**Figure 2A**; **Supplementary Figures 1A-C**). By the end of the follow-up, 112 deaths occurred: 38 in the T group, 44 in the TL group, and 30 in the TLT group. The TLT group showed a significantly longer OS (median, 31.5 [95% CI, 27.8-NA] months) compared with the T (median, 18.5 [95% CI, 10.6-23.0] months; *P*<0.001) and TL (median, 20.5 [95% CI, 15.7-30.2] months; *P*=0.013) groups (**Figure 2B**; **Supplementary Figures 1D-F**).

**Figure 3:**
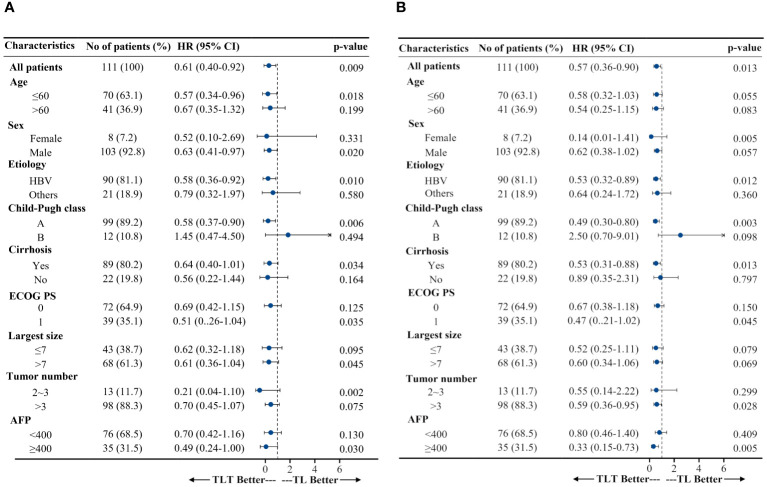
Subgroup analyses of progression-free survival **(A)** and overall survival **(B)**. HR, hazard ratio; CI, confidence interval; ECOG PS, Eastern Cooperative Oncology Group performance status; T, TACE, transarterial chemoembolization; TL, TACE combined with lenvatinib; TLT, TACE combined with lenvatinib and tislelizumab; AFP, α-fetoprotein.

After adjusting for the baseline patient characteristics, multivariable Cox regression analyses revealed that the treatment regimen was independently associated with PFS and OS. Specifically, for PFS, the adjusted hazard ratios (HRs) were 0.60 (95% CI, 0.37-0.96; *P*=0.034) for the TL group and 0.35 (95% CI, 0.22-0.56; *P*<0.001) for the TLT group vs. TACE monotherapy. For OS, the HRs were 0.64 (95% CI, 0.41-1.00, *P*=0.051) for the TL group and 0.37 (95% CI, 0.23-0.60; *P*<0.001) for the TLT vs. TACE monotherapy (**Supplementary Table 1**). Subgroup analyses highlighted that the TLT group consistently demonstrated superior PFS and OS compared with the TL group in most subgroups defined by baseline patient characteristics, except for the Child-Pugh B subgroup (**Figure 3**).

### 3.4 Progression pattern and subsequent treatments

There were no statistically significant differences in the patterns of disease progression, including local lesion progression, intrahepatic metastasis, extrahepatic metastasis, or death, among the three groups (*P*=0.055). The T group had numerically higher proportions of local lesion progression (36.8% vs. 24.4% vs. 21.1%) and intrahepatic metastasis (42.1% vs. 31.1% vs. 22.8%) compared with the TL and TLT groups. The TLT group had the lowest local lesion progression and intrahepatic metastasis proportions among the three groups (**Supplementary Table 2**).

After tumor progression, most patients received subsequent antitumor treatment: 80.0% from the T group, 78.4% from the TL group, and 83.7% from the TLT group. A combination of TACE with TKIs was the most frequent subsequent treatment in the T group, accounting for 39.4%. In addition, the proportion of TACE combined with MTAs and ICIs was 14.3%, and no patients chose hepatic artery infusion chemotherapy (HAIC) combined with MTAs and ICIs or TACE combined with HAIC and MTAs and ICIs. The patients in the TL group predominantly favored a regimen of TACE in combination with MTAs and ICIs at 27.6%, and 20.7% opted for TACE plus HAIC in combination with MTAs and ICIs. Meanwhile, the patients in the TLT group mostly opted for TACE plus HAIC in combination with MTAs and ICIs and HAIC in combination with MTAs and ICIs, representing 27.8% and 22.2%, respectively (**Supplementary Table 3**).

### 3.5 TRAEs

The TRAEs were primarily related to the TACE procedure and are listed in **Table 3**. The most common TRAEs were aminotransferase increased, abdominal pain, fever, and nausea; most were moderate in severity. The incidence of grade 3 or 4 TRAEs was higher in the TLT and TL groups compared with the T group. AEs resulting in dose reduction or interruption of lenvatinib or tislelizumab were observed in eight (17.8%) patients in the TL group and 11 (16.7%) patients in the TLT group. These AEs were manageable, and no AEs leading to permanent treatment discontinuation or treatment-related death were reported during the study period at the four participating centers.

**Table 3:** Treatment-related adverse events.

Event, n (%)	Any grade			*P*	Grade 3/4			*P*
	T (n=38)	TL (n=45)	TLT (n=66)		T (n=38)	TL (n=45)	TLT (n=66)	
Abdominal pain	15 (39.5)	21 (46.7)	30 (45.5)	0.812	2 (5.3)	4 (8.9)	5 (7.6)	0.363
Nausea	11 (28.9)	18 (40.0)	24 (36.4)	0.789	1 (2.6)	2 (4.4)	2 (3.0)	0.286
Diarrhea	5 (13.2)	17 (37.8)	29 (43.9)	0.339	0 (0)	5 (11.1)	8 (12.1)	–
Fever	15 (39.5)	18 (40.0)	28 (42.4)	0.636	0 (0)	1 (2.2)	2 (3.0)	–
Aminotransferase increased	30 (78.9)	38 (84.4)	55 (83.3)	0.685	5 (13.2)	15 (33.3)	21 (31.8)	0.809
Hypothyroidism	0 (0)	8 (17.8)	17 (25.8)	–	0 (0)	2 (4.4)	3 (4.5)	–
Platelet count decreased	3 (7.9)	11 (24.4)	19 (28.8)	0.809	0 (0)	3 (6.7)	5 (7.6)	–
Hypertension	0 (0)	6 (18.8)	8 (37.5)	–	0 (0)	0 (0)	0 (0)	–
Hand-foot syndrome	0 (0)	11 (24.4)	18 (27.3)	–	0 (0)	3 (6.7)	4 (6.1)	–
Proteinuria	0 (0)	9 (20.0)	13 (19.7)	–	0 (0)	3 (6.7)	3 (4.5)	–
Bleeding (gingiva)	0 (0)	4 (8.9)	6 (9.1)	–	0 (0)	1 (2.2)	1 (1.5)	–
Immune-related AEs	NA	NA	17 (25.8)	–	NA	NA	3 (4.5)	–

T, TACE, transarterial chemoembolization; TL, TACE combined with lenvatinib; TLT, TACE combined with lenvatinib and tislelizumab; NA, not applicable.

## 4 Discussion

The present study was the first to evaluate the effectiveness and safety of TACE plus TKIs and PD-1 inhibitors for BCLC-B HCC beyond the up-to-11 criteria, compared with TACE plus TKIs and TACE monotherapy. The present study displays several innovative points, such as the high tumor burden (i.e., beyond the up-to-11 criteria), the inclusion of patients with intermediate-stage HCC (which display high heterogeneity), comparison among three treatments, all three treatments are first-line standard regimens for HCC. Significant ORR, PFS, and OS improvements were observed with TACE plus lenvatinib and tislelizumab. Subgroup analyses further echoed these findings, consistently indicating superior survival outcomes across the subgroups, all converging in favor of the triple combination therapy. Although the TLT group reported a slightly higher incidence of TRAEs than the T and TL groups, most of these events were mild-to-moderate and manageable.

For patients with intermediate-stage HCC, complete ORR and PFS data are lacking in the published literature for the subgroup of patients with HCC beyond the up-to-11 criteria. A recent retrospective study showed that the CR rate was 38.7% in patients with intermediate-stage HCC beyond up-to-11 criteria (28). Previous research reported that in patients with BCLC-B HCC beyond the up-to-7 criteria, TACE monotherapy induced an ORR of 33.3% and a median PFS of 3.0 months (29). These findings align well with the outcomes of the present study, where the T group showed an ORR of 31.6% and a median PFS of 4.4 months. The results strongly suggest that not all patients benefit from TACE; such patients are defined as “TACE-refractory” and “TACE-unsuitable” (30). New treatment strategies, such as early initiation of systemic therapies, have been recommended in such patients (31). In the present study, the survival benefit of the TL group was better than that of the T group (TL vs. T, median OS: 20.5 vs. 18.5 months; median PFS: 5.5 vs. 4.4 months). Beyond the up-to-11 criteria (HR=1.694, P<001) was reported to be an independent predictor of OS in BCLC-B HCC (13). In addition, a previous study showed that the median OS of TACE monotherapy was 11.3 months in patients with BCLC-B with HCC beyond the up-to-11 criteria (12). Studies by Kudo et al. (29) and Tada et al. (32) also revealed that in patients with unresectable BCLC-B HCC beyond the up-to-7 criteria, those who initially received lenvatinib had superior prognosis to those administered TACE monotherapy. It suggests that TACE in combination with lenvatinib may have a more pronounced beneficial effect than TACE monotherapy, particularly in patients bearing a high tumor burden.

Despite the potential of TACE in combination with lenvatinib, the prognosis of patients with unresectable BCLC-B HCC beyond the up-to-11 criteria may remain suboptimal due to high tumor burden. A previous investigation by the authors in patients with unresectable HCC highlighted the synergistic benefits of combining TACE with lenvatinib and pembrolizumab, leading to significant improvements in OS (median, 18.1 vs. 14.1 months) and PFS (median, 9.2 vs. 5.5 months) compared with TACE plus lenvatinib (33). The CHANCE001 trial reported the superior prognosis of TACE combined with PD-(L) 1 inhibitors and MTAs over TACE monotherapy (median OS: 19.2 vs. 15.7 months; median PFS: 9.5 vs. 8.0 months) in a cohort of patients (predominantly Chinese) with advanced HCC (34). The EMERALD-1 trial (BCLC-A, -B, and -C stages) showed that TACE combined with durvalumab and bevacizumab improved PFS compared with TACE in patients with unresectable HCC (15.0 vs. 8.2 months, P=0.032) (35). Previous trials also supported the use of tislelizumab in advanced HCC (18) and the use of lenvatinib in such patients (36, 37). The LEAP-002 trial supports the combination of lenvatinib plus pembrolizumab vs. lenvatinib alone (PFS of 8.2 vs. 8.0 months) (19), while the CARES-310 trial supports the use of an ICI with a TKI in advanced HCC (PFS of 5.6 months vs. 3.7 months with sorafenib) (20). Furthermore, recent retrospective analyses underscored the survival benefits of a TACE-lenvatinib-PD-(L)1 inhibitor regimen vs. the TACE-lenvatinib combination in patients with advanced or unresectable HCC (38, 39). Regarding the mechanism by which lenvatinib enhances the efficacy of immunotherapy, many basic studies have already explored and elucidated. Chen, et al. reported that lenvatinib inhibited the FGFR4 signaling pathway, downregulated the expression of PD-L1 on tumor cells, and limited the differentiation of Tregs, thereby modulating the tumor immune microenvironment to enhance the efficacy of PD-1 (40). Deng, et al. reported that both of vascular endothelial growth factor (VEGF) and fibroblast growth factor (FGF) increased in tumor and suppressed the immune microenvironment, lenvatinib can reduce the level of these two cytokines to improve the efficacy of PD-1 (41). Besides, as reported, TACE also has the function of remodeling the tumor immune microenvironment to improve the efficacy of PD-1 (42, 43). In total, TACE administrated in combination with systemic therapy-based treatment offers a new paradigm for unresectable HCC, including intermediate stage beyond up-to-11 criteria (44, 45). Notably, the present study suggested a numerically longer median OS with the triple combination therapy (31.5 months) compared with previous studies. This discrepancy can be attributed mainly to the patient pool; while earlier studies predominantly encompassed BCLC-C HCC patients, the present study targeted those in the BCLC-B stage. Moreover, the median PFS remained relatively consistent across different studies exploring the triple combination therapy, suggesting a more pronounced enhancement in OS than PFS across different HCC stages.

The advantages of combining TACE with lenvatinib and tislelizumab remained broadly consistent across a variety of clinical subgroups compared with the TACE-lenvatinib combination, including the subgroups relevant to HCC prognosis, such as age, sex, etiology, baseline tumor burden, and α-fetoprotein (AFP) levels. In addition, for BCLC-B HCC patients with Child-Pugh A, TACE with lenvatinib and tislelizumab resulted in better PFS and OS than TACE with lenvatinib. As is well known, the magnitude of tumor burden may be quite heterogeneous in the BCLC-B stage. The prognosis is also influenced by AFP concentration and the degree of liver function impairment, even if it still belongs to Child-Pugh class A (4, 46, 47). Elevated AFP values predict a higher risk of HCC recurrence and, thus, lower survival (4). Repeated TACE interventions may compromise liver function, consequently influencing patient survival (48). Of interest, the present study also found that for patients with AFP ≥400, TACE with lenvatinib and tislelizumab resulted in better PFS and OS than TACE-lenvatinib, and TACE with lenvatinib and tislelizumab was superior in PFS to TACE-lenvatinib for patients with tumors >7 cm in diameter. In addition, the combination therapy could reduce the number of TACE sessions in this study (six, four, and three in the T, TL, and TLT groups, respectively), probably contributing to better liver function reserve. It suggests a promising efficacy advantage for TACE with lenvatinib and tislelizumab. Further prospective studies are needed to confirm these findings.

Local lesion progression and intrahepatic metastasis can limit the survival benefit conferred by TACE (49). In the present study, the proportions of local lesion progression and intrahepatic metastasis were the highest in the T group and the lowest in the TLT group. After progression, over 75% of patients in each group received subsequent antitumor treatment. TACE or/and HAIC plus MTAs and ICIs were administered to 66.7% of patients in the TLT group, compared with 55.2% in the TL group and 14.3% in the T group. PFS (8.5 vs. 5.5 vs. 4.4 months) and OS (31.5 vs. 20.5 vs. 18.5 months) were significantly longer in the TLT group compared with the TL group and T group. These results suggest that combining TACE with lenvatinib and tislelizumab could effectively control local disease progression and improve the survival benefit. It can be speculated that local therapies induce antigen and proinflammatory cytokine release, whereas VEGF inhibitors and tyrosine kinase inhibitors boost immunity and prime tumors for checkpoint inhibition (50). Hence, combining TACE with lenvatinib and tislelizumab could provide a synergistic antitumor effect.

Regarding safety, the TLT group exhibited a higher incidence of overall and grade 3-4 TRAEs, particularly immune-related AEs. This trend aligns with prior expectations, as previous clinical trials examining the combination of immunotherapy and targeted therapy have reported elevated incidences of grade ≥3 AEs, i.e., 61.6% in IMbrave 150 and 56% in ORIENT-32 (21, 51). In addition, the incidence of aminotransferase elevations in grade 3-4 TRAEs was higher in the TL group than in the TLT and T groups (33.3% vs. 31.8% vs. 13.2%), which is similar to the safety finding in the LAUNCH trial (52). Most AEs were mild-to-moderate in severity and either readily manageable or reversible in this study without affecting subsequent treatments.

Although favorable therapeutic responses and survival were observed in the present cohort, this study had limitations. First, the retrospective study nature may have induced biases. Second, although both lenvatinib monotherapy and tislelizumab monotherapy are recommended in guidelines for treating HCC, their combination remains outside standard recommendations and needs further investigation. Third, the sample size was relatively small, and the follow-up period was relatively short. Hence, future large-scale prospective studies are warranted to verify these findings.

Compared with TACE monotherapy and TACE plus lenvatinib, the combination of TACE, lenvatinib, and tislelizumab showed significantly improved ORR, PFS, and OS in patients with BCLC-B HCC beyond the up-to-11 criteria with an acceptable safety profile. Therefore, this triple combination therapy could be a potential superior treatment option for these patients. RCTs should be performed to confirm the results.

## Data availability statement

The original contributions presented in the study are included in the article/**Supplementary Material**. Further inquiries can be directed to the corresponding author.

## Ethics statement

The studies involving humans were approved by ethics committee of the First Affiliated Hospital of Sun Yat-sen University (#2021-782). The studies were conducted in accordance with the local legislation and institutional requirements. The participants provided their written informed consent to participate in this study.

## Author contributions

SC: Conceptualization, Data curation, Formal Analysis, Methodology, Writing – original draft, Writing – review & editing. TS: Data curation, Formal Analysis, Writing – original draft, Writing – review & editing. FS: Data curation, Formal Analysis, Writing – original draft, Writing – review & editing. HJC: Data curation, Formal Analysis, Writing – original draft, Writing – review & editing. ZW: Data curation, Formal Analysis, Writing – original draft, Writing – review & editing. LW: Data curation, Formal Analysis, Writing – original draft, Writing – review & editing. PM: Data curation, Formal Analysis, Writing – original draft, Writing – review & editing. YZ: Conceptualization, Data curation, Formal Analysis, Methodology, Writing – original draft, Writing – review & editing. QM: Data curation, Formal Analysis, Writing – original draft, Writing – review & editing. FW: Data curation, Formal Analysis, Writing – original draft, Writing – review & editing. JL: Data curation, Formal Analysis, Writing – original draft, Writing – review & editing. XC: Conceptualization, Data curation, Formal Analysis, Methodology, Writing – original draft, Writing – review & editing. HWC: Conceptualization, Data curation, Formal Analysis, Methodology, Writing – original draft, Writing – review & editing. WG: Conceptualization, Data curation, Formal Analysis, Methodology, Writing – original draft, Writing – review & editing.
