# Supplementary material for: TACE plus lenvatinib and tislelizumab for intermediate-stage hepatocellular carcinoma beyond up-to-11 criteria: a multicenter cohort study
Source: Front Immunol. 2024 Jul 26;15:1430571. doi: 10.3389/fimmu.2024.1430571 (PMC11310062; doi:10.3389/fimmu.2024.1430571)
Supplement: Supplementary file 1 [file DataSheet_1.docx]

***Supplementary materials***

**TACE plus lenvatinib and tislelizumab for intermediate-stage hepatocellular carcinoma beyond up-to-eleven criteria: A multicenter cohort study**


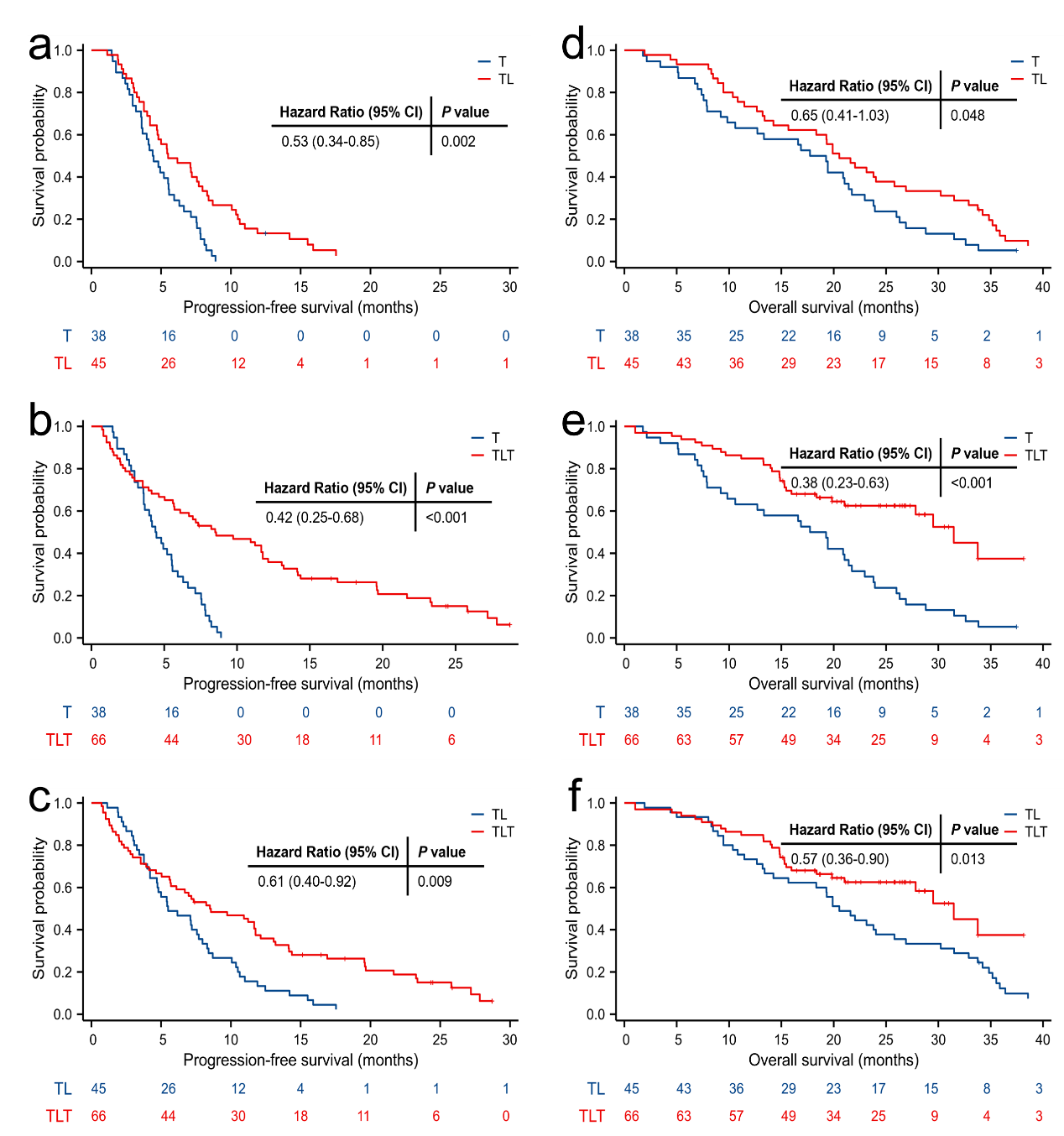


**Figure S1.** **Kaplan-Meier analysis of progression-free survival (a-c) and overall survival (d-f) in groups T vs. TL, T vs. TLT, and TL vs. TLT.** T, TACE, Transarterial Chemoembolization; TL, TACE combined with lenvatinib; TLT, TACE combined with lenvatinib and tislelizumab.

**Table S1.** Factors associated with progression-free survival and overall survival

| **Characteristics** | **Univariate** | | **Multivariate** | |
| --- | --- | --- | --- | --- |
|  | **HR (95% CI)** | ***P*** | **HR (95% CI)** | ***P*** |
| **PFS** |  |  |  |  |
| Age (≤60 vs. >60) | 0.79 (0.56 - 1.12) | 0.186 |  |  |
| Sex (female vs. male) | 0.70 (0.49 - 1.01) | 0.052 | 0.80 (0.54 - 1.19) | 0.276 |
| Child-Pugh class (A vs. B) | 0.68 (0.41 - 1.13) | 0.154 |  |  |
| Number of tumors (2-3 vs. >3) | 0.65 (0.38 - 1.11) | 0.092 | 0.67 (0.39 - 1.16) | 0.153 |
| Tumor size (>7 vs. ≤7) | 1.34 (0.95 - 1.89) | 0.101 |  |  |
| AFP (≥400 vs. <400) | 1.26 (0.89 - 1.80) | 0.201 |  |  |
| Cirrhosis (yes vs. no) | 1.80 (0.53 - 2.12) | 0.281 |  |  |
| Etiology (others vs. HBV) | 1.16 (0.74 - 1.82) | 0.522 |  |  |
| Treatment |  |  |  |  |
| T | Reference |  | Reference |  |
| TL | 0.55 (0.35 - 0.87) | **0.010** | 0.60 (0.37 - 0.96) | **0.034** |
| TLT | 0.32 (0.20 - 0.50) | **<0.001** | 0.35 (0.22 - 0.56) | **<0.001** |
| **OS** |  |  |  |  |
| Age (≤60 vs. >60) | 1.03 (0.70 - 1.51) | 0.879 |  |  |
| Sex (female vs. male) | 0.77 (0.51 - 1.17) | 0.216 |  |  |
| Child-Pugh class (A vs. B) | 0.59 (0.35 - 1.01) | 0.072 | 0.73 (0.43 - 1.27) | 0.268 |
| Number of tumors (2-3 vs. >3) | 0.90 (0.50 - 1.65) | 0.735 |  |  |
| Tumor size (>7 vs. ≤7) | 1.21 (0.82 - 1.78) | 0.348 |  |  |
| AFP (≥400 vs. <400) | 1.26 (0.86 - 1.86) | 0.239 |  |  |
| Cirrhosis (yes vs. no) | 1.29 (0.82 - 2.04) | 0.281 |  |  |
| Etiology (others vs. HBV) | 1.07 (0.66 - 1.74) | 0.796 |  |  |
| Treatment |  |  |  |  |
| T | Reference |  | Reference |  |
| TL | 0.63 (0.41 - 0.98) | **0.039** | 0.64 (0.41 - 1.00) | 0.051 |
| TLT | 0.35 (0.22 - 0.57) | **<0.001** | 0.37 (0.23 - 0.60) | **<0.001** |

Notes: The multivariable analysis includes the variables with *P*≤0.10 from the univariable analyses.

Abbreviations: HR, hazard ratio; CI, confidence intervals; PFS, progression-free survival; HBV, hepatitis B virus; T, TACE, transarterial chemoembolization; TL, TACE combined with lenvatinib; TLT, TACE combined with lenvatinib and tislelizumab; AFP, α-fetoprotein; OS, overall survival.

**Table S2.** Progression-free survival (PFS) events in the three groups

| **PFS event, n (%)** | **T (n=38)** | **TL (n=45)** | **TLT (n=57)** | ***P*** |
| --- | --- | --- | --- | --- |
| Local lesion progression | 14 (36.8) | 11 (24.4) | 12 (21.1) | 0.055 |
| Intrahepatic metastasis | 16 (42.1) | 14 (31.1) | 13 (22.8) |  |
| Extrahepatic metastasis | 5 (13.2) | 12 (26.7) | 18 (31.6) |  |
| Death | 3 (7.9) | 8 (17.8) | 14 (24.5) |  |

Abbrevations: T, TACE, transarterial chemoembolization; TL, TACE combined with lenvatinib; TLT, TACE combined with lenvatinib and tislelizumab.

**Table S3.** Subsequent treatment after tumor progression

| **Subsequent treatment, n (%)** | **T (n=35)** | **TL (n=37)** | **TLT (n=43)** |
| --- | --- | --- | --- |
| Antitumor treatment | 28 (80.0) | 29 (78.4) | 36 (83.7) |
| TKIs | 3 (10.7) | 3 (10.3) | 0 (0) |
| TACE + TKIs | 11 (39.4) | 3 (10.3) | 0 (0) |
| TACE + MTAs + ICIs | 4 (14.3) | 8 (27.6) | 6 (16.7) |
| Ablation | 2 (7.1) | 0 (0) | 0 (0) |
| MTAs + ICIs | 0 (0) | 2 (6.9) | 7 (19.4) |
| HAIC | 2 (7.1) | 0 (0) | 0 (0) |
| HAIC + TKIs | 0 (0) | 4 (13.8) | 1 (2.8) |
| HAIC + MTAs + ICIs | 0 (0) | 2 (6.9) | 8 (22.2) |
| TACE+HAIC | 4 (14.3) | 0 (0) | 0 (0) |
| TACE + HAIC + TKIs | 2 (7.1) | 1 (3.5) | 4 (11.1) |
| TACE + HAIC + MTAs + ICIs | 0 (0) | 6 (20.7) | 10 (27.8) |
| Best support care | 7 (20.0) | 8 (21.6) | 7 (16.3) |

Notes: TKIs include lenvatinib, sorafenib, regorafenib, and apatinib.

MTAs plus ICIs include TKIs plus PD-1/PD-L1/CTLA-4 inhibitors or anti-VEGF antibodies plus PD-1/PD-L1 inhibitors.

Abbrevations: T, TACE, transarterial chemoembolization; TL, TACE combined with lenvatinib; TLT, TACE combined with lenvatinib and tislelizumab; HAIC, hepatic artery infusion chemotherapy (FOLFOX); TKIs, tyrosine kinase inhibitors; MTAs, molecular targeted agents; ICIs, Immune checkpoint inhibitors; PD-1, programmed death 1; PD-L1, programmed death-ligand 1; CTLA-4, cytotoxic T lymphocyte-associated antigen-4; VEGF, vascular endothelial growth factor.
